# Supplementary material for: Bridging glucose metabolism and intrinsic functional organization of the human cortex
Source: Commun Biol. 2026 Feb 10;9:377. doi: 10.1038/s42003-026-09693-w (PMC12992575; doi:10.1038/s42003-026-09693-w)
Supplement: Supplementary file 2 — Supplemental Information [file 42003_2026_9693_MOESM2_ESM.pdf]

# Supplementary materials for

## "Bridging Glucose Metabolism and Intrinsic Functional Organization of the Human Cortex"

Bin Wan [1,2,3 *], Valentin Riedl [4,5], Gabriel Castrillon [4,5], Matthias Kirschner [3,6], Sofie L. Valk [1,2,7 *]

1. *Lise Meitner Research Group Cognitive Neurogenetics, Max Planck Institute for Human Cognitive and Brain Sciences, Leipzig, Germany.*
2. *Institute of Neuroscience and Medicine (INM-7: Brain and Behavior), Research Center Jülich, Jülich, Germany.*
3. *Department of Psychiatry, University Hospitals of Geneva, Geneva, Switzerland.*
4. *Department of Neuroradiology at Klinikum rechts der Isar, TUM School of Medicine and Health, Technical University of Munich, Munich, Germany.*
5. *Department of Neuroradiology at Uniklinikum Erlangen, Friedrich-Alexander-University Erlangen-Nuremberg, Erlangen, Germany.*
6. *Synapsy Center for Neuroscience and Mental Health Research, University of Geneva, Geneva, Switzerland.*
7. *Institute of Systems Neuroscience, Heinrich Heine University Düsseldorf, Düsseldorf, Germany.*

* Correspondence to Bin Wan (binwan@cbs.mpg.de) and Sofie L. Valk (valk@cbs.mpg.de), Otto Hahn Group Cognitive Neurogenetics, Max Planck Institute for Human Cognitive and Brain Sciences, Leipzig, Germany; Institute of Neuroscience and Medicine (INM-7: Brain and Behavior), Research Centre Jülich, Jülich, Germany.

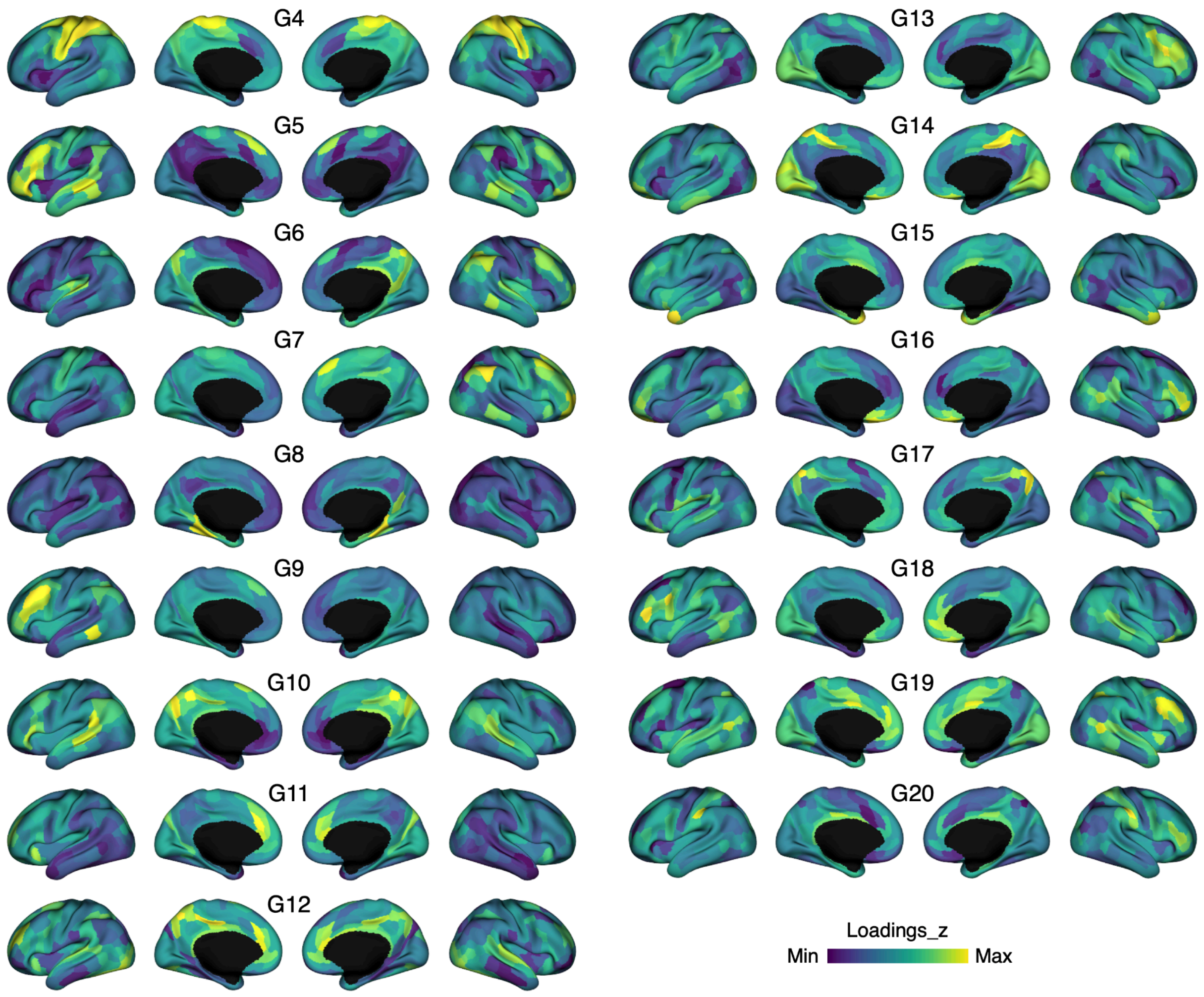


**Figure S1**. G4-20 of the group-level functional connectome.

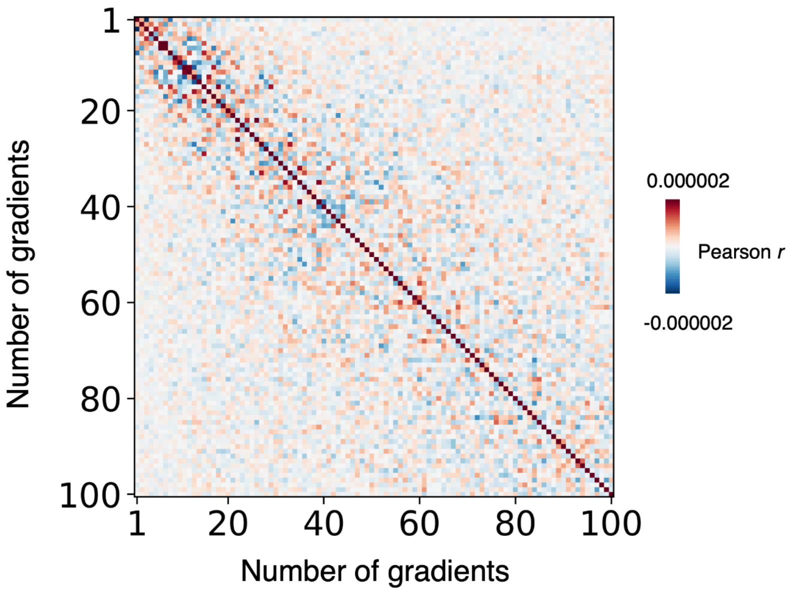


**Figure S2**.Inter-gradient correlation to identify the independence of each gradient.

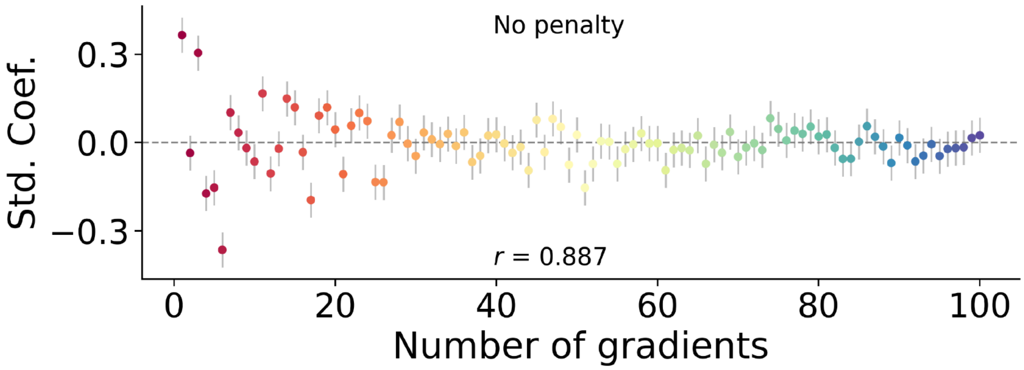


**Figure S3**. Standardized regression coefficient and 95% confidence interval for non-penalty model.

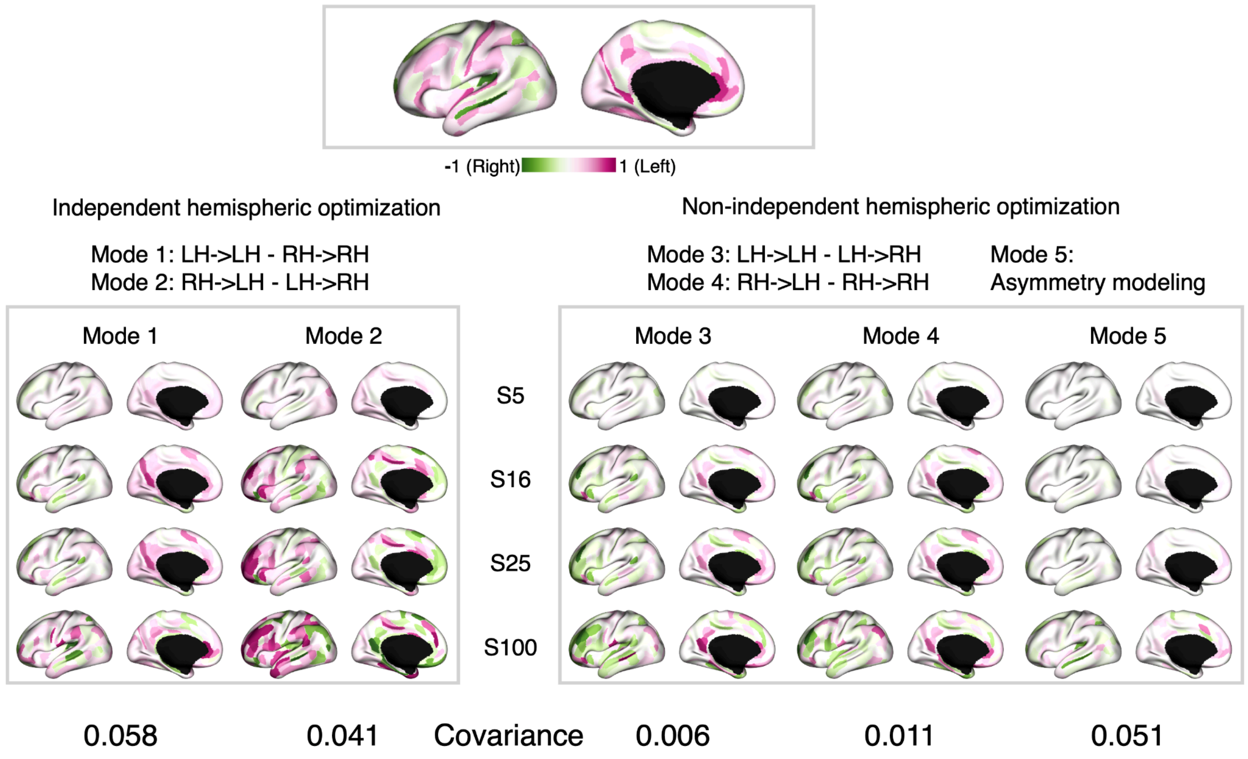


**Figure S4**. Competition versus lateralization using different modes.

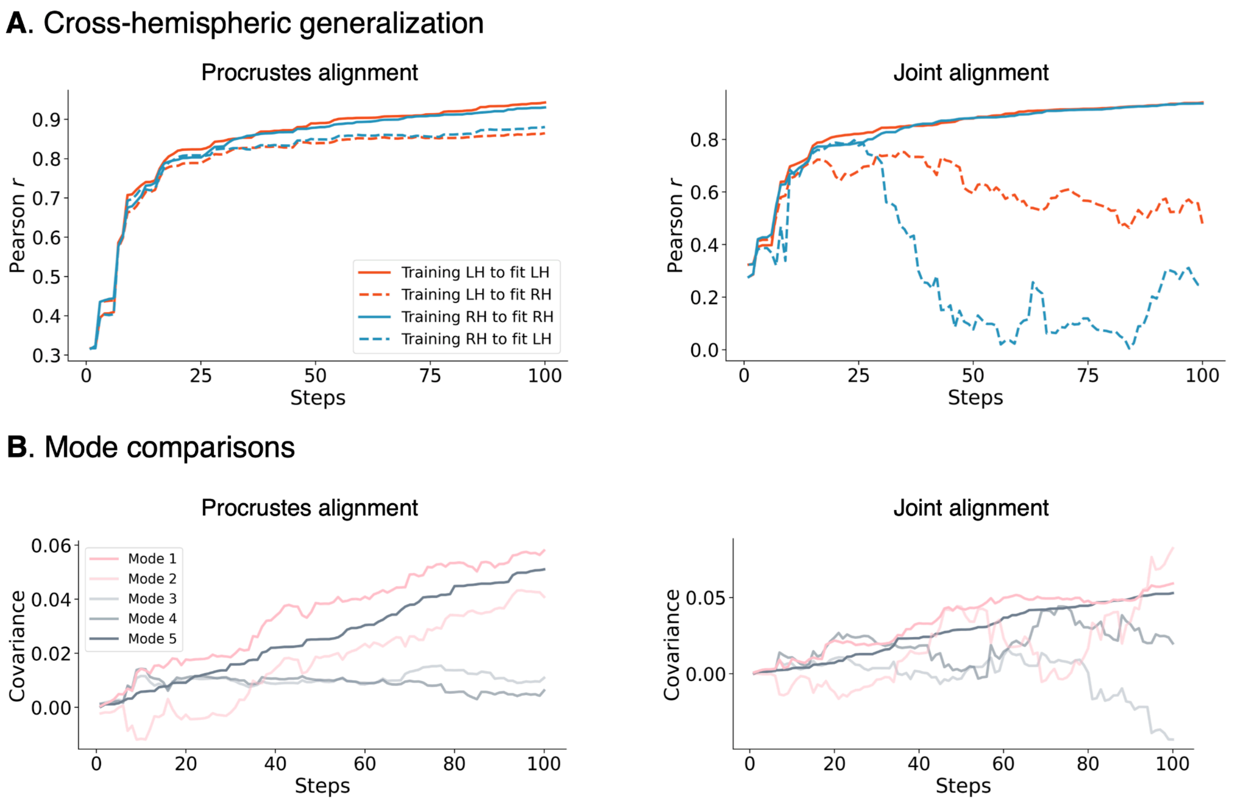


**Figure S5**. Replication analysis using joint alignment between left and right hemispheres. **A**. The cross-hemispheric generalization corresponding to **Figure 5C**. We used gradients after joint alignment to perform this analysis. **B**. Mode comparisons corresponding to **Figure 5F**. It compares fitting covariance between the specific (modes 1 and 2, pink curves) and generalization (modes 3, 4, and 5, grey curves) models.

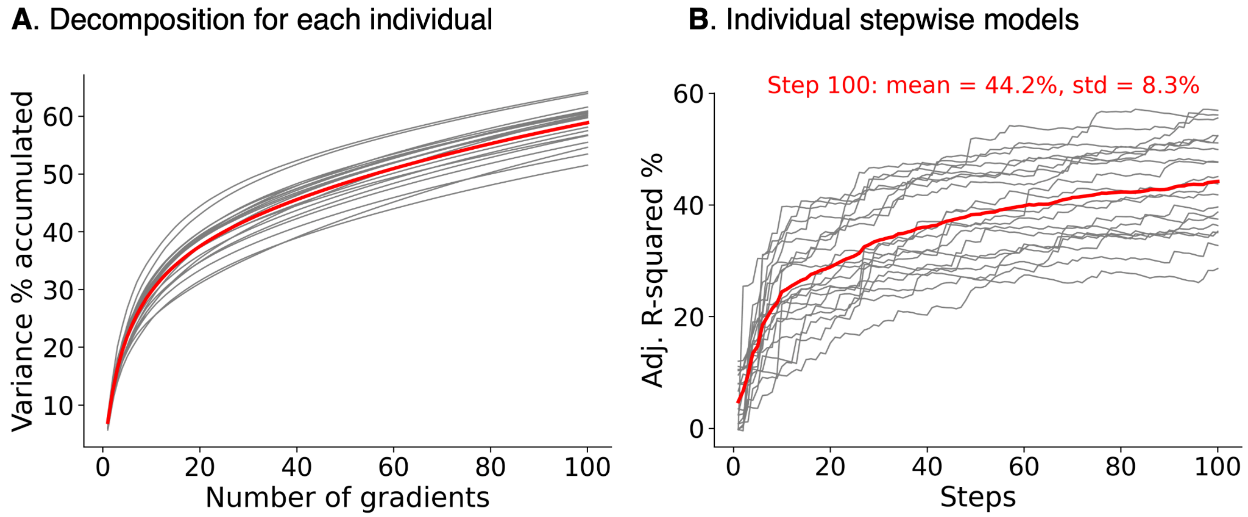


**Figure S6**. Individual analyses (sparsity = 0.9). **A**. The accumulative lambda values for the decomposition. **B**. Individual stepwise models. Gray and red lines indicate individual level curve and mean across individuals.

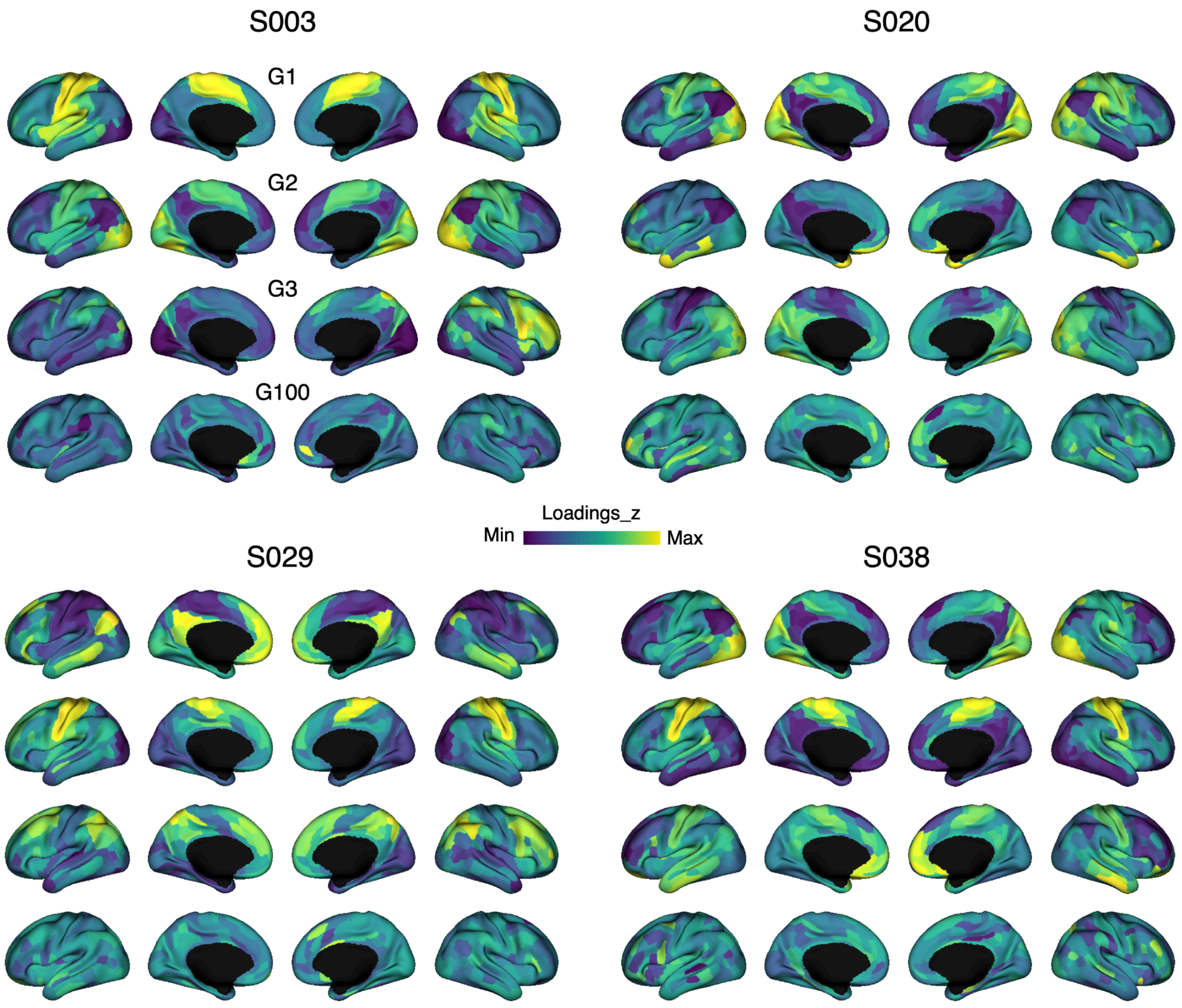


**Figure S7**. Raw gradient loading maps (G1-3 and G100) for four subjects (S003, S020, S029, and S038) .

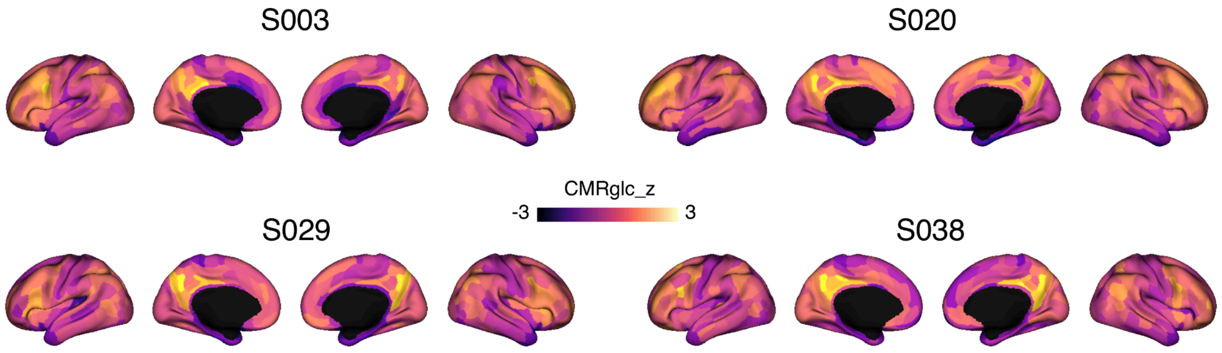


**Figure S8**. Raw glucose metabolism map (CMRglc) for four subjects(S003, S020, S029, and S038) .

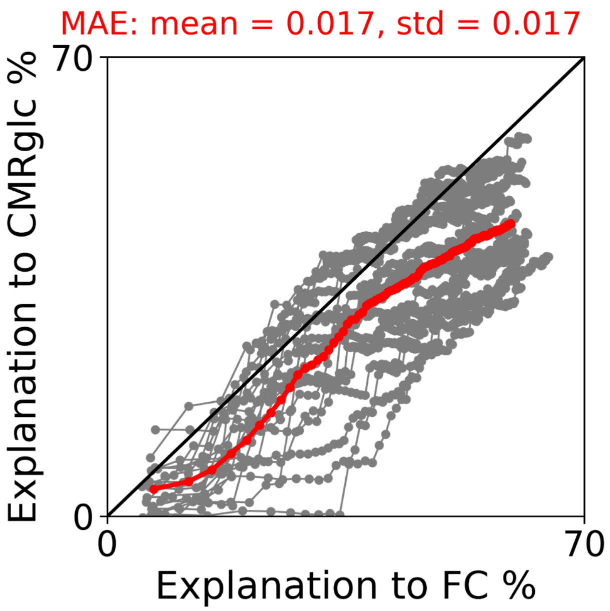


**Figure S9**. Gradient explanation to FC and CMRglu map at individual level. Gray and red lines indicate individual level curve and mean across individuals.

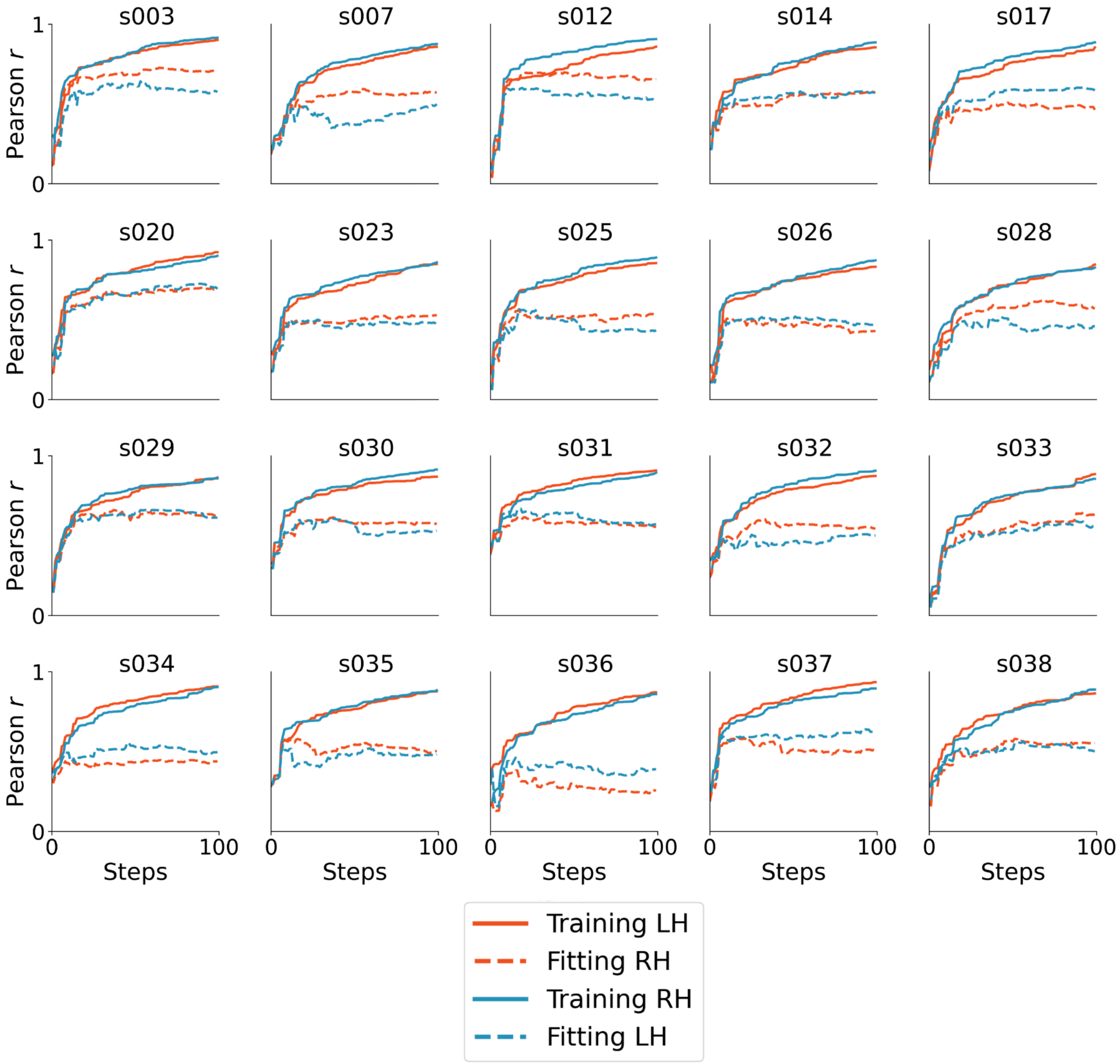


**Figure S10**. Cross-hemispheric validation for each individual.

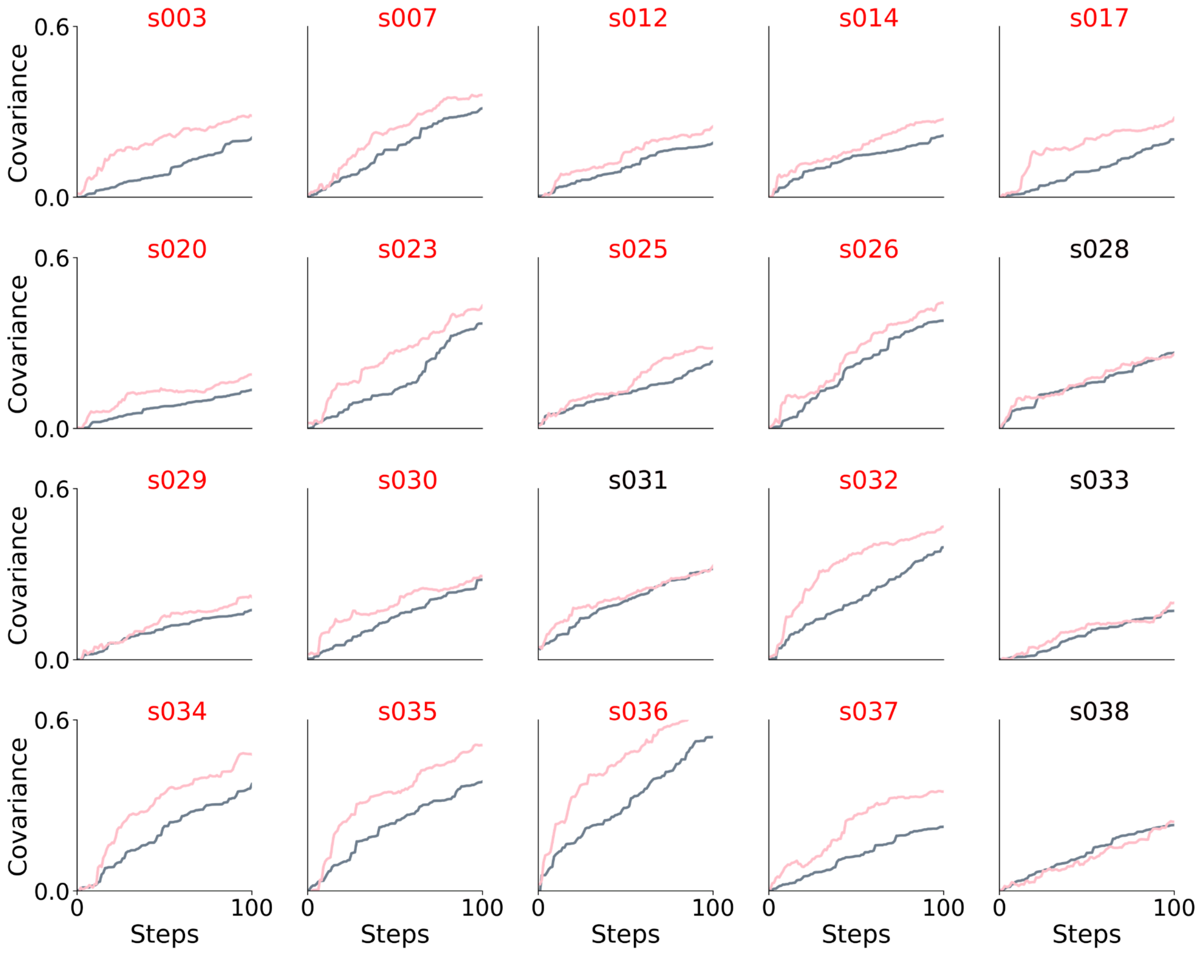


**Figure S11**. Competition versus lateralization for each individual. Red and black titles indicate individuals with clear and unclear comparison.

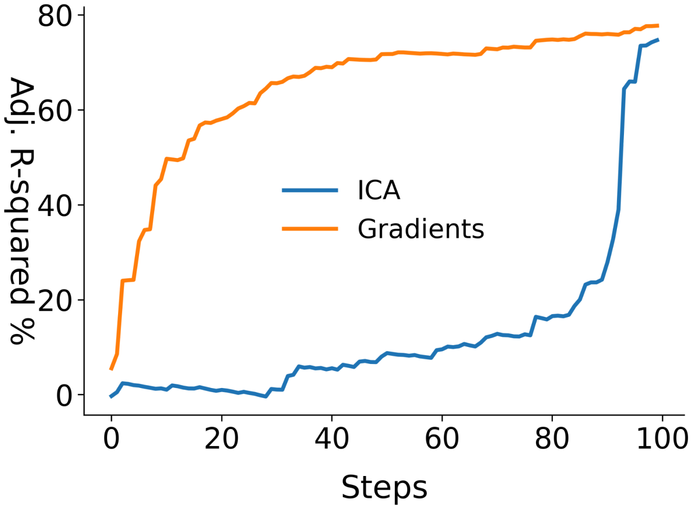


**Figure S12**. Comparisons between using gradient and independent components, supplementary to **Fig. 2B**.
